# Fetal aneuploidy screening by non-invasive prenatal testing of maternal plasma DNA sequencing with “false negative” result due to confined placental mosaicism: A case report

**DOI:** 10.1097/MD.0000000000020848

**Published:** 2020-07-17

**Authors:** Xiaozhou Li, Duan Ju, Yunfang Shi, Yan Li, Haiwei Dong, Jianhua Huang, Ying Zhang

**Affiliations:** Tianjin Prenatal Diagnosis Center, Department of Gynecology and Obstetrics, Tianjin Medical University General Hospital, Anshan Street 154#, Heping District, Tianjin, China.

**Keywords:** non-invasive prenatal testing, trisomy 18, mosaicism, double aneuploidy, amniocentesis

## Abstract

**Rationale:**

Non-invasive prenatal testing (NIPT) is an accurate screening method with high specificity and sensitivity and a low false-positive rate of trisomy 21, 18, and 13. However, false-negative NIPT results could also limit the clinical application of NIPT.

**Patient concerns:**

A 34-year-old primigravida woman who underwent NIPT at 16 + 3 weeks’ gestation was identified as being at high risk for fetal trisomy X (47, XXX). Fetal cardiac defect and hand posture were observed during prenatal ultrasound examination at the 23rd week of gestation.

**Diagnoses:**

Amniocentesis conducted at the 24th week of gestation. Fetal karyotyping and FISH identified karyotype 48, XXX, + 18, which indicated that the NIPT failed to detect trisomy 18 in this case.

**Interventions:**

The couple decided to terminate pregnancy at the 26th week of gestation and was willing to undergo further examinations.

**Outcomes:**

Discordant results between fetus with trisomy 18 and placenta with mosaic T18 were further identified with massive parallel sequencing, which might be due to that the fetal cell-free DNA in maternal plasma for NIPT that was assessed principally originated from the trophoblast cells.

**Lessons:**

The presence of trisomy 18 mosaicism in the placenta might be the reason for the false-negative NIPT result in this case of double aneuploidy with 48, XXX, + 18, karyotype. Although the NIPT is a valuable screening method that has evident advantages in prenatal aneuploidy screening for certain chromosomal abnormalities compared to other methods, it is not a “diagnostic test” yet.

## 1 Introduction

Non-invasive prenatal testing (NIPT) for common fetal aneuploidies by massive parallel sequencing of maternal plasma cell-free DNA is an accurate screening method with high specificity and sensitivity and with low false-positive rates.^[1,2]^ The weighted pooled detection rates of trisomy 13, 18, and 21 in singleton pregnancies were 99.7%, 97.9%, and 99.0%, respectively.^[3]^ However, discordant results between amniocentesis and NIPT were observed in several studies.^[2]^ There was also no evidence whether NIPT could be used to detect double aneuploidy in the same individual. The cases of double aneuploidy 48, XXY, + 21 and 48, XXX, + 18 had mostly been reported.^[4]^ We presented here a case of double aneuploidy 48, XXX, + 18, which was only diagnosed positive for trisomy 18 by NIPT.

## 2 Case presentation

Test data were collected from of a 34-year-old gravida 1, para 0 (G1P0) pregnant woman who underwent NIPT through massive parallel sequencing at 16 + 3 weeks’ gestation due to advanced maternal age at delivery with normal parental ultrasound examination result. Maternal plasma cell-free DNA was extracted from 8 mL maternal peripheral blood and sequenced using Illumina HiSeq2000 platform.^[5]^ This study was performed with the approval of Medical Ethics Committee of Tianjin Medical University General Hospital (the reference number is IRB2019-WZ-053) and written informed consent was obtained from the patient.

**Figure 1:**
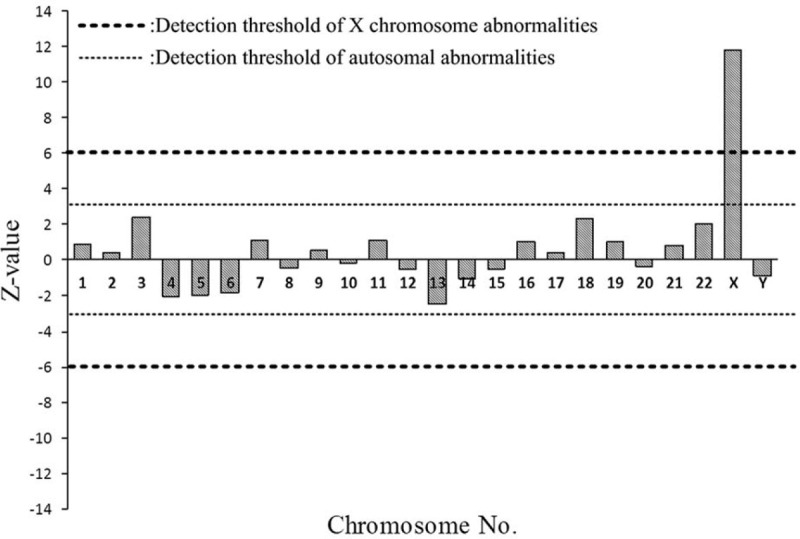
Z-value of NIPT results.

**Figure 2:**
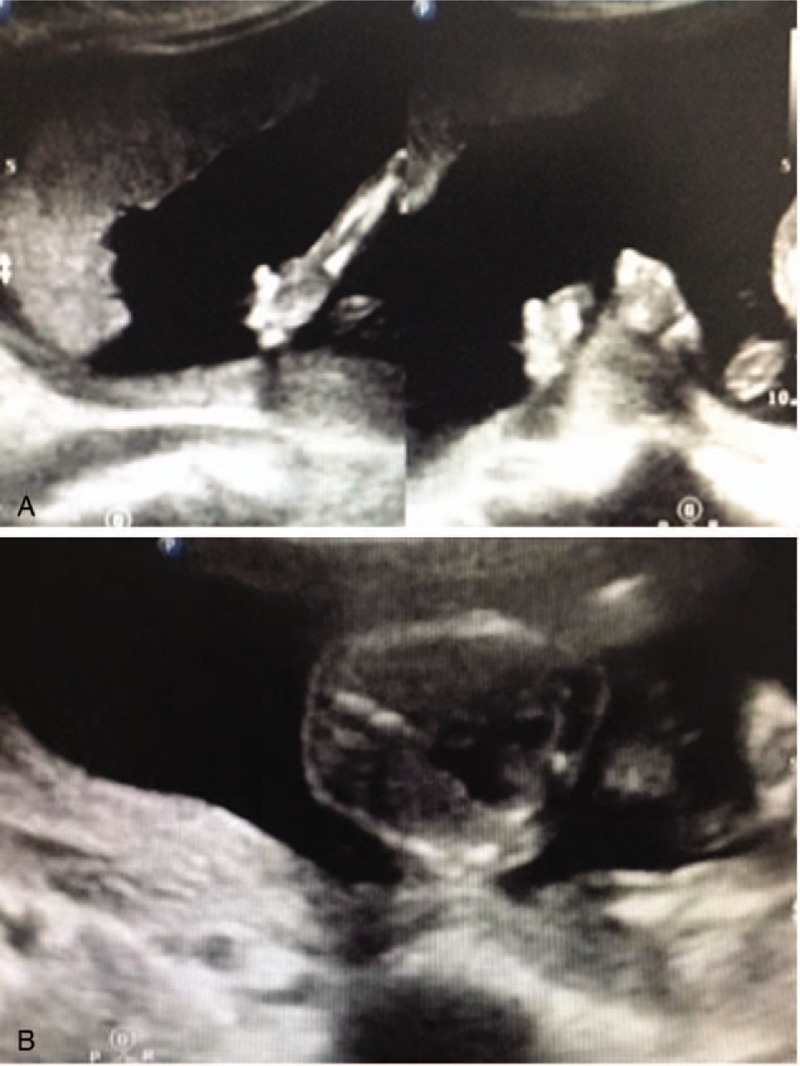
Conventional karyotype analysis of cultured amniocytes. A: The figure of karyotype analysis. B: The figure of cell division phase.

**Figure 3:**
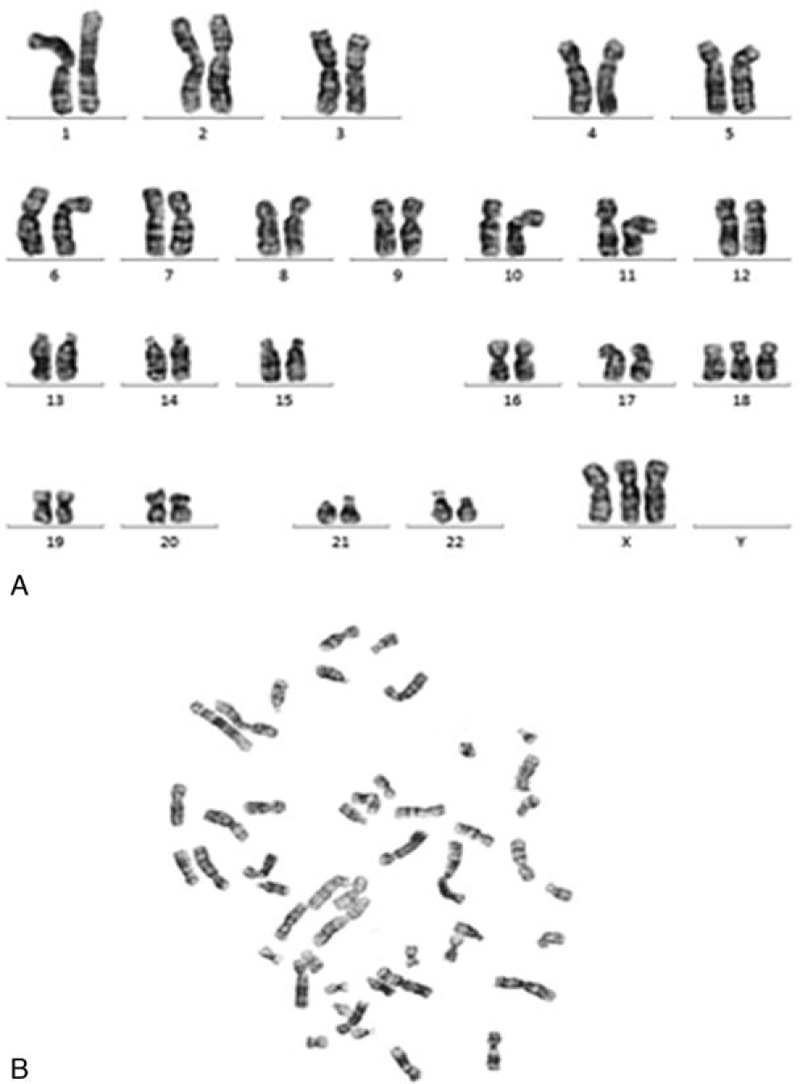
FISH analysis of amniocytes. The chromosome 18 was marked as green (G) and the chromosome X was marked as blue (B). The cells with karyotype 48, XXX, + 18 were indicated as 3G3B.

**Figure 4:**
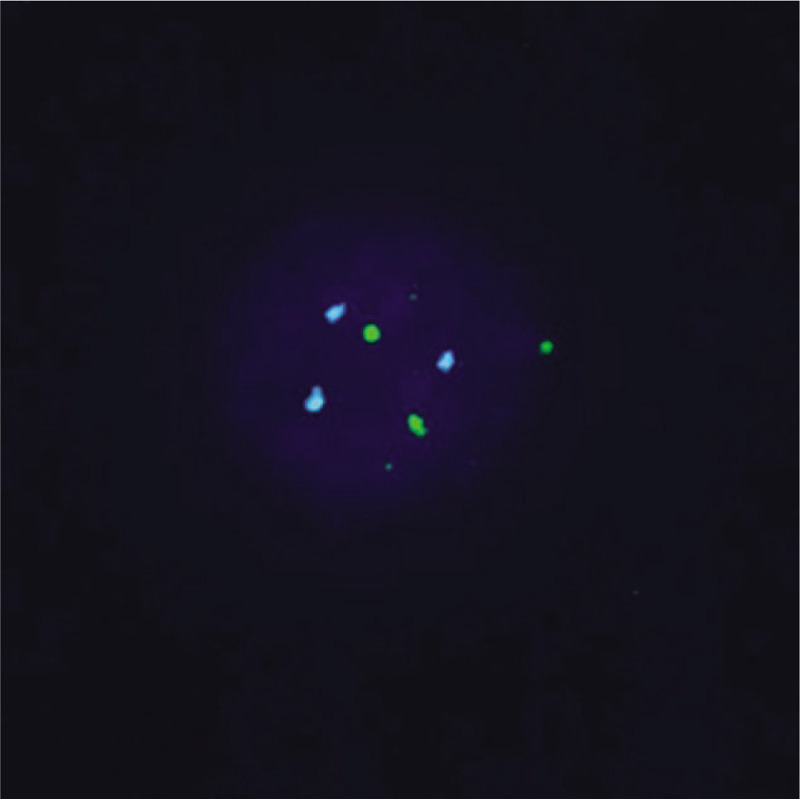
Abnormal sonographic features of fetus at 23th week of gestation. A: Overriding fingers. B: Atrioventricular septal defect.

**Figure 5:**
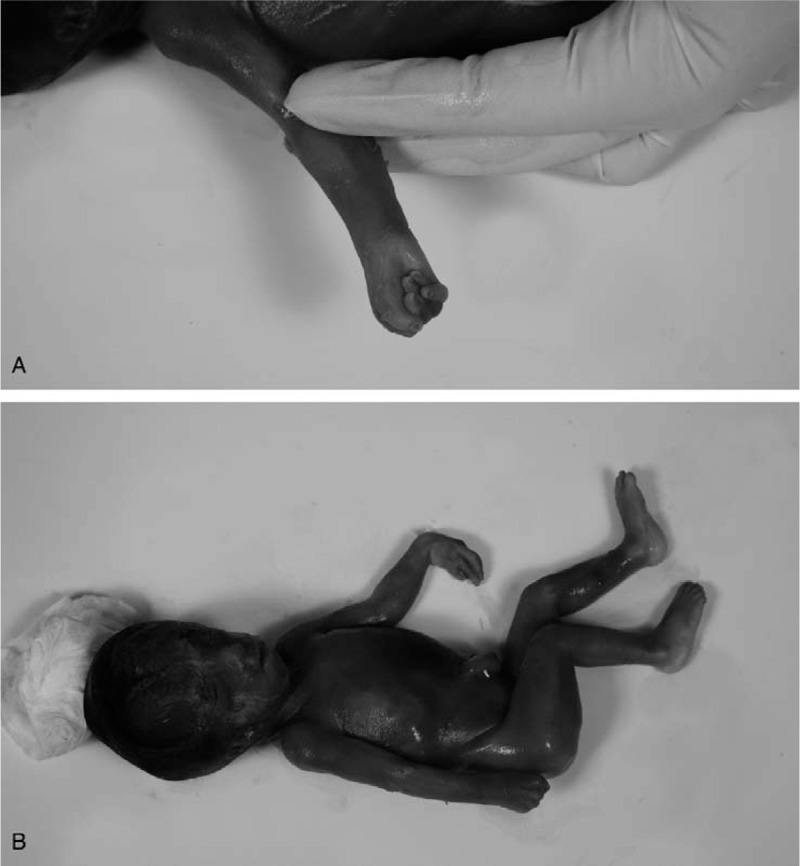
The induced abortion fetus. A: Whole body of induced abortion fetus. B: Distinctive hand posture of induced abortion fetus.

**Figure 6:**
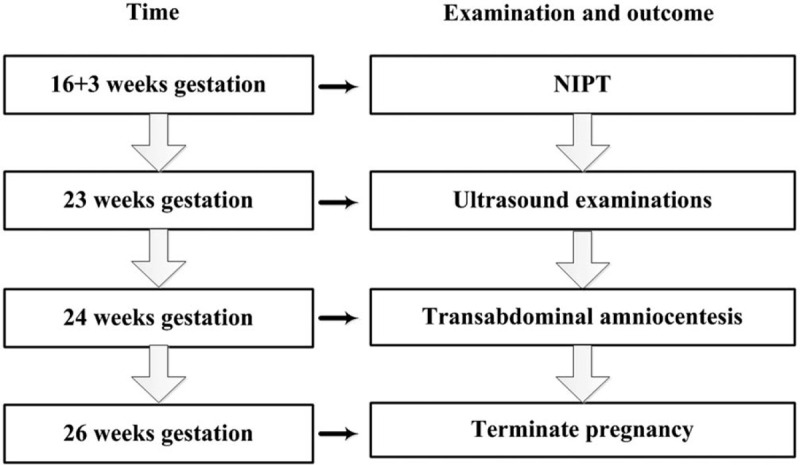
The time line of this case report.

The NIPT results revealed that this pregnant woman had a fetus at high risk of trisomy X (47, XXX) but at low risk of trisomy 13, 18, and 21. The total read of massive parallel sequencing result was 5.01 Mb, and the uniMap read was 3.17 Mb. The Z-value of NIPT results for chromosome 18 was 2.29, but for chromosome X, it was approximately 11.79 (Fig. 1), which suggested the fetus had a high risk of trisomy X (47, XXX). After genetic counseling, the patient did not want to undergo a further invasive prenatal diagnostic test because the couple was willing to raise a female baby with 47, XXX. Subsequently, the pregnant woman underwent a systemic ultrasound examination at 23 weeks of gestation. Atrioventricular septal defect and distinctive hand posture (overriding fingers) were detected in the fetus by ultrasound examinations (Fig. 2). After consulting the woman at 24 weeks of gestation, a transabdominal amniocentesis was performed. Abnormal 48, XXX, + 18 karyotypes were identified by G-banding analysis of cultivated amniocytes (Fig. 3). Trisomy X and trisomy 18 were all also detected by fluorescence in situ hybridization (FISH) with chromosome 18- and X-specific probes (Fig. 4). The results of fetal karyotyping and FISH implied that the NIPT might have revealed a false-negative result of trisomy 18. The couple decided to terminate pregnancy at the 26th week of gestation and was willing to undergo further examinations. As shown in Figure 5, the features of the aborted fetus were in accordance with the typical features of a fetus with trisomy 18. The maternal and fetal parts of the placenta and different segments of the umbilical cord were sampled after induced labor. Genome-wide screening for copy number variations was performed on all the samples. The results showed that trisomy X was found in all the samples, while trisomy 18 was found with a mosaic ratio of approximately 40% in the placenta samples, and trisomy 18 was found in all segments of the umbilical cord samples (Table 1). The time line of this case report was summarized in Figure 6.

**Table 1:**
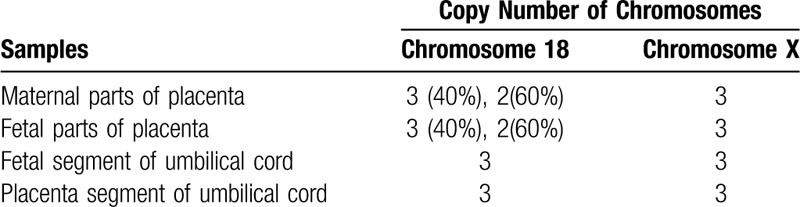
The copy number variations results of placental DNA.

## 3 Discussion

NIPT showed broad application prospects for prenatal screening of common fetal autosomal aneuploidies.^[6]^ In China, standard prenatal aneuploidy screening with serum markers was performed in all pregnant women. NIPT was performed on pregnant women who were at high risk with common fetal autosomal aneuploidies by serum screening after 12 weeks’ gestation according to the technical specification of NIPT in China. However, fetal cell-free DNA in maternal peripheral blood was considered to originate primarily from placental cytotrophoblast and syncytiotrophoblast cells; thus, fetal cell-free DNA mainly consisted of placental cell-free DNA fragments. Therefore, the karyotype discrepancy between fetal and placental tissue may affect the NIPT results and lead to inaccurate conclusions. Confined placental mosaicism (CPM) is considered to account for the phenomenon in which abnormal cell lines can be detected frequently in placenta samples but cannot be detected in fetal tissue. In approximately 2% of viable pregnancies, CPM is detected through chorionic villus sampling at 9 to 11 weeks of gestation.^[7]^ CPM usually caused false-positive NIPT results. Hence, a cytogenetic abnormality, most often trisomy, is found to be confined to the placenta and absent in the fetus.^[8]^ However, it will cause false-negative NITP results when cytogenetic abnormality is confined to the fetus but mosaicism is confined to the placenta. In this case, because of CPM, the fraction of fetal cell-free DNA for chromosome 18 was considerably lower than the detection threshold of NIPT test for trisomy 18 and consequently caused false-negative result. Therefore, the level of mosaicism is an important factor that can influence the accuracy of NIPT.

False-negative results have been reported and noted in recent years, although NIPT tests relatively show a low level of false-negative results. For example, Wang et al^[9]^ reported a case of a positive NIPT result of trisomy 5, but negative NIPT result of trisomy 18, that was detected in a pregnant woman, while only trisomy 18 was observed in the fetal karyotyping analysis after amniocentesis. Pan et al^[10]^ reported a case of a negative NIPT result of trisomy 18 that was detected in a pregnant woman; however, trisomy 18 was observed in the fetal karyotyping analysis. Published studies also reported that the rate of false-negative results of NIPT for trisomy 18 was higher than that for trisomy 21 and trisomy 13.^[11]^ Trisomy 18 syndrome is the second most common autosomal aneuploidy syndrome after trisomy 21 syndrome in liveborn infants.^[12]^ Individuals with trisomy 18 syndrome present with multisystem alterations, specifically the malformations of the circulatory and skeletal system, which could often be detected by ultrasound examination. In this case, the heart malformations and hand malformations were observed in the fetus, which further strengthened the evidence obtained by invasive prenatal diagnosis.

## 4 Conclusion

The results of fetal karyotyping and FISH implied that the NIPT might have revealed a false-negative result of trisomy 18. Although the NIPT is a valuable screening method that has evident advantages in prenatal aneuploidy screening for certain chromosomal abnormalities compared to other methods, it is not a “diagnostic test” yet.

## Acknowledgments

We would like to thank Editage (www.editage.cn) for English language editing.

## Author contributions

**Conceptualization:** Xiaozhou Li, Duan Ju and Yunfang Shi.

**Data curation:** Xiaozhou Li and Duan Ju.

**Investigation:** Yan Li and Haiwei Dong.

**Methodology:** Yan Li and Haiwei Dong.

**Project administration:** Jianhua Huang and Ying Zhang.

**Supervision:** Jianhua Huang and Ying Zhang.

**Writing – original draft:** Xiaozhou Li.

**Writing – review &editing:** Jianhua Huang and Ying Zhang.

## References

[1] Mackie F Hemming K Allen S The accuracy of cell-free fetal DNA-based non-invasive prenatal testing in singleton pregnancies: a systematic review and bivariate meta-analysis. BJOG 2017;124:32–46. 27245374 10.1111/1471-0528.14050

[2] Gregg AR Skotko BG Benkendorf JL Noninvasive prenatal screening for fetal aneuploidy, 2016 update: a position statement of the American College of Medical Genetics and Genomics. Genet Med 2016;18:1056–65. 27467454 10.1038/gim.2016.97

[3] Gil MM Accurti V Santacruz B Analysis of cell-free DNA in maternal blood in screening for aneuploidies: updated meta-analysis. Ultrasound Obstet Gynecol 2017;50:302–14. 28397325 10.1002/uog.17484

[4] Liao F Huynh D Bhatt S Clinical experience with non-invasive prenatal testing (NIPT) for rare autosomal trisomies. Ultrasound Obstet Gynecol 2018;52 (S1):221–321.28976617

[5] Liang D Lv W Wang H Non-invasive prenatal testing of fetal whole chromosome aneuploidy by massively parallel sequencing. Prenat Diagn 2013;33:409–15. 23299662 10.1002/pd.4033

[6] Bianchi DW Chiu RW Sequencing of circulating cell-free DNA during pregnancy. N Engl J Med 2018;379:464–73. 30067923 10.1056/NEJMra1705345 PMC10123508

[7] Kalousek DK Vekemans M Confined placental mosaicism. J Med Genet 1996;33:529–33. 8818935 10.1136/jmg.33.7.529 PMC1050657

[8] Mennuti MT Cherry AM Morrissette JJD Is it time to sound an alarm about false-positive cell-free DNA testing for fetal aneuploidy? Am J Obstet Gynecol 2013;415–9. 2013/11/01/. 23529082 10.1016/j.ajog.2013.03.027

[9] Wang Z Tang X Yang S A gradual change of chromosome mosaicism from placenta to fetus leading to T18 false negative result by NIPS. Clin Chim Acta 2019;495:263–8. 30998911 10.1016/j.cca.2019.04.064

[10] Pan M Li FT Li Y Discordant results between fetal karyotyping and non-invasive prenatal testing by maternal plasma sequencing in a case of uniparental disomy 21 due to trisomic rescue. Prenat Diagn 2013;33:598–601. 23533085 10.1002/pd.4069

[11] Grati FR Malvestiti F Ferreira JCPB Fetoplacental mosaicism: potential implications for false-positive and false-negative noninvasive prenatal screening results. Genet Med 2014;16:620–4. 24525917 10.1038/gim.2014.3

[12] Hui L Slonim DK Wick HC Novel neurodevelopmental information revealed in amniotic fluid supernatant transcripts from fetuses with trisomies 18 and 21. Hum Genet 2012;131:1751–9. 22752091 10.1007/s00439-012-1195-x PMC3472090

